# Exploring oxylipins in processed foods: Understanding mechanisms, analytical perspectives, and enhancing quality with lipidomics

**DOI:** 10.1016/j.heliyon.2024.e35917

**Published:** 2024-08-09

**Authors:** Putri Widyanti Harlina, Vevi Maritha, Xiang Yang, Roy Dixon, Muchtaridi Muchtaridi, Raheel Shahzad, Ernisa Adha Nur’Isma

**Affiliations:** a Department of Food Industrial Technology, Faculty of Agro-Industrial Technology, Universitas Padjadjaran, 45363, Bandung, Indonesia; b Department of Pharmaceutical Analysis and Medicinal Chemistry, Faculty of Pharmacy, Universitas Padjadjaran, 45363, Bandung, Indonesia; c Department of Animal Science, University of California Davis, California, 95616, United States; d Department of Chemistry, California State University, Sacramento, CA, 95819, United States; e Research Center for Genetic Engineering, National Research and Innovation Agency (BRIN), Indonesia; f Pharmacy Study Program, Faculty of Health and Science, Universitas PGRI Madiun, Indonesia

**Keywords:** Oxylipins, Processed food, Lipid compound, Lipidomics, PUFAs

## Abstract

Oxylipins are active lipid compounds formed through the oxidation of unsaturated fatty acids. These compounds have drawn considerable attention due to the potential impact on human health and processed food quality. Therefore, this study aimed to deepen current understanding and assess recent analytical advancements regarding the physiological roles of oxylipins in processed food products using lipidomics. The mechanisms behind oxylipins production in processed foods were extensively investigated, underscoring potential associations with chronic diseases. This indicates the need for innovative strategies to mitigate harmful oxylipins levels to enhance the safety and shelf life of processed food products. The results showed that mitigation methods, including the use of antioxidants and optimization of processing parameters, reduced oxylipins levels. The integration of lipidomics with food safety and quality control processes is evident in cutting-edge methods such as nuclear magnetic resonance and mass spectrometry for compliance and real-time evaluation. Aside from envisioning the future trajectory of food science and industry through prospective studies on oxylipins and processed foods, the results also provide the basis for future investigations, innovation, and advancements in the dynamic field of food science and technology.

## Highlights

• Bioactive oxylipins' influence on food quality and human health scrutinized.
• Advances in lipidomics enhance comprehension of oxylipins in processed foods.
• Mitigation strategies, such as antioxidants and optimized processing, to reduce detrimental oxylipins.
• Cutting-edge lipidomics methods (NMR, mass spectrometry) for real-time assessment, ensuring food safety.

## 1 Introduction

The consumption of processed food products in contemporary diets is a fundamental aspect that significantly influences dietary patterns [1]. The variety of food products including pre-prepared meals and packed snacks has significantly transformed consumption approach, providing numerous advantages, such as convenience, prolonged shelf life, and a diverse selection of appealing flavors [2]. Processed foods are common in contemporary societies as packaged products available in supermarket aisles, which cater to the demands of hectic lifestyles and fast-paced routines [3].

The content and functioning of processed food products are significantly influenced by the diverse group of organic molecules known as lipids [4,5]. The incorporation of these essential fatty acids is crucial in the creation of a wide range of food items, as it enhances not only the flavor and texture but also the nutritional content and overall quality. In the modern food industry, lipids are employed for many different things, such as making candies softer and making fat-soluble vitamins more soluble [6]. By changing the lipid content or adding chemicals, the compounds control the quality of food. Lipids and additives in food products interact, changing the product's quality. Moreover, the modification of the lipid composition affects the food's overall quality by changing the nutritional makeup. Numerous molecular pathways, such as the generation of free radicals and reactive oxygen precursors, play a role in oxidation. This mechanism influences how food ingredients interact, resulting in the creation of both desirable and undesirable molecules. Oxidation, which occurs during manufacture, storage, distribution, and final preparation for consumption, is a major cause of deterioration for food lipids [6].

In general, not only are lipids essential for eating, but are also important for many other facets of human health. Excessive intake of certain fats has been linked to negative health consequences such as obesity and heart disease. However, these substances support cellular processes that are essential to health by preserving the strength and rigidity of cell membranes [7]. This emphasizes how important it is to maintain an appropriate balance of fats in daily food consumption to support the achievement of peak physiological function [8,9]. The intricate interactions among diverse lipid constituents, such as unsaturated fats, saturated fats, and essential fatty acids, greatly impact inflammatory responses, cholesterol levels, and the structure of cellular membranes [10,[11], [13]]. These standards bear the greatest significance in the assessment of general health and state of well-being.

Oxylipins are a class of lipid-derived substances functioning as signaling molecules that include metabolites and oxygenated fatty acids [14]. In the dimension of processed food products, these substances have drawn interest. Different bioactive compounds are produced when polyunsaturated fatty acids (PUFAs) and monounsaturated fatty acids (MUFAs) experience oxidation. These molecules contain many different chemical structures and a broad spectrum of biological functions, according to Misheva et al. [15] and Dyall et al. [16]. For instance, oxylipins are associated with inflammatory disorders such as Type 2 diabetes, obesity, and NAFLD (non-alcoholic fatty liver disease) [15]. Further studies on the possible impacts of processed food on quality, safety, and nutritional value have been prompted by the functions in numerous physiological systems linked to human health [17].

Over the past few years, oxylipins have become increasingly recognized as important markers for assessing lipid degradation and rancidity progression in the processed foods industry [18,19]. Püssa et al. [124] explored the impact of lipid oxidation on food quality and safety, emphasizing the role of oxylipins as indicators. Jackson and Penumetcha [125] also investigated the health effects of consuming oxidized lipids, providing insights into the associated risks and mitigation strategies. In addition, Kozłowska et al. [126] analyzed the effectiveness of different antioxidants in preventing lipid oxidation in processed foods. Moreover, the analysis provides options for developing functional foods with enhanced nutritional benefits because specific oxylipins possess anti-inflammatory and antioxidant properties [20,21]. This study was conducted to present a comprehensive review regarding oxylipins and the potential applications in processed foods. The results provide insightful information about how these compounds may affect the food industry as well as possible health and safety concerns for consumers.

The advantages of processed foods, including convenience, flavor appeal, and longer shelf life, have a significant impact on modern eating habits [2]. However, the number of bioactive ingredients present in these products poses possible adverse health effects [3]. It is necessary to understand the role of oxylipins in processed foods to assess the impact on human health. According to Yuan et al. [22], lipidomics is a highly efficient analytical method that permits the comprehensive identification and quantification of lipids, including oxylipins, inside complex food matrices. Targeted lipidomics provides a high-throughput, very sensitive method for the comprehensive and effective quantification of oxylipins in biological and clinical samples [23]. Therefore, this study aimed to present a thorough investigation and analysis regarding the role of oxylipins in processed food items using lipidomics method. The intricate relationship between lipid oxidation and oxylipins synthesis in various processed food products was evaluated using this cutting-edge technology.

## 2 Oxylipins and lipidomics overview

### 2.1 Definition and characteristics of oxylipins

Oxylipins are oxygenated metabolites derived from fatty acids through a carefully controlled mechanism and carry out various functions, such as signaling and transportation [24]. These groups of compounds, which can also be generated through autooxidation, are produced when unsaturated fatty acids experience spontaneous reaction with oxygen in the presence of heat or light [18]. Autooxidation is the process in which unsaturated fatty acids interact with air, resulting in the formation of reactive oxygen species. These species can then interact with the double bonds in fatty acids, leading to the generation of different oxidation products, such as oxylipins [25].

**Fig. 1:**
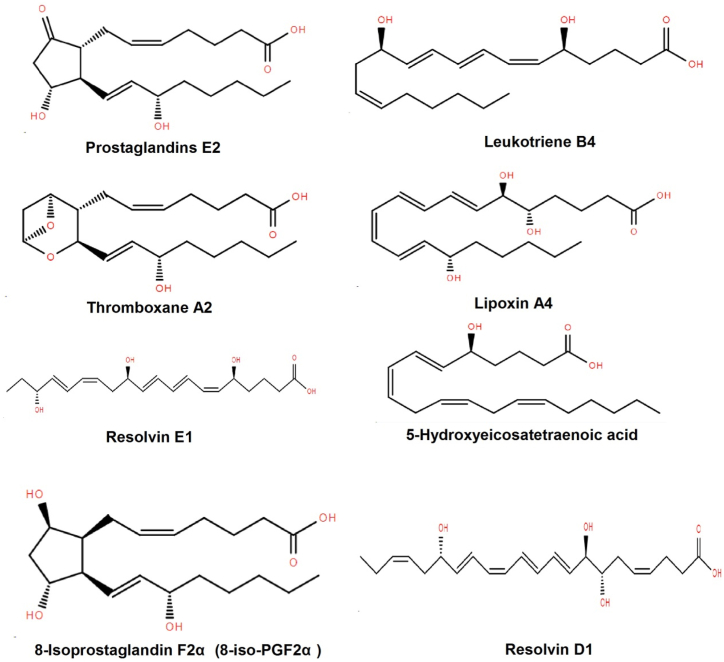
Diverse group of oxylipins derived from the oxidation of polyunsaturated fatty acids (PUFAs).

As mediators of cellular signaling, inflammation, and responses to oxidative stress in plant and animal organisms, oxylipins are crucial to many biological processes [15,20,26]. The scientific term "oxylipins," formed from the combination of "oxygenated lipids," describes a large range of lipid-derived compounds produced through the enzymatic or non-enzymatic oxidation of PUFAs and MUFAs. These compounds include Docosahexaenoic acid (DHA), linoleic acid (LA), and arachidonic acid (AA). The enzymatic conversion of PUFAs into oxylipins is aided by three primary mechanisms, namely cyclooxygenase (COX), lipoxygenase (LOX), and cytochrome P450 (CYP) pathways [27,28]. Each pathway produces different oxylipins (Fig. 1), which can be distinguished by the stereochemistry and location of the oxygenated functional groups. The structural diversity, comprising isoprostanes, complicated epoxy-fatty acids, and simple hydroxy-fatty acids can be used to explain the oxidative metabolism of PUFAs [29]. The location and stereochemistry of the oxygenated functional groups determine the specific biological activity connected to each class of oxylipins. These behaviors contribute to the functions as effective mediators of cellular responses and communication [30].

A significant way by which oxylipins regulate inflammation is through the synthesis of prostaglandin, a byproduct of COX pathway. Several studies have demonstrated the importance of these molecules for immunological responses, vasodilation, and pain regulation [31,32]. F2-isoprostanes also provide valuable information about lipid peroxidation and the connection to several disorders. These compounds serve as biomarkers of lipid peroxidation, and the assessment offers a reliable approach to assessing food quality control. Furthermore, F2-isoprostanes are a group of biologically active substances that function as dependable indicators for lipid peroxidation, namely the process of breaking down PUFAs in cell membranes and lipoproteins by oxidation [33]. These compounds are produced by non-enzymatic peroxidation of arachidonic acid, which is an omega-6 PUFA present in cell membranes [34,35]. Additionally, molecules generated from lipids such as F2-isoprostanes are significant indicators of oxidative stress [[36], [37], [38], [39], [40]]. Due to the complex nature, the assessment of oxylipins can provide insight into lipid biochemical and physiological processes. This presents several exciting opportunities for studies into the various roles in human health and disease management.

### 2.2 Lipidomics is a potent analytical method for studying lipids, particularly oxylipins

Human health has been the subject of several studies on oxylipins, especially as a biomarker for cancer, heart disease, and inflammation [41]. Even though these substances are found in food naturally, processing and storage might raise the levels, which could result in increased exposure [42,43]. Enzymatic reactions that take place during various food preparation phases, such as frying, baking, roasting, and preservation, as well as lipid oxidation mechanisms can provide oxylipins [[44], [45], [46]]. Lipidomics method provides a strong way to perform a comprehensive analysis of oxylipins and derivatives, which helps to improve current understanding of the presence, distribution, and possible health effects in processed foods [[47], [48], [49]]. Table 1 presents lipidomics methods for oxylipin analysis in food quality control.

**Table 1:** Lipidomics approach for oxylipins analysis in food quality control.

No	Author	Title	Oxylipins and derived	Characteristics and Function	Equipment	Results	Food Products	Ref
1	Medina, S. et al.	Quantification of phytoprostanes – bioactive oxylipins – and phenolic compounds of *Passiflora edulis* Sims shell using UHPLC-QqQ-MS/MS and LC-IT-DAD-MS/MS	Phytoprostanes	Phytoprostanes are not essential for the metabolic activity of living cells but they are considered components of oxidative injury-sensing systems and act as excellent biomarkers of oxidative degradation	UHPLC-QqQ-MS/MS	Six new bioactive oxylipins –phytoprostanes – were detected in gulupa shell by a UHPLC-QqQ-MS/MS method: F1t-phytoprostanes and D1t-phytoprostanes were the predominant and minor classes, respectively	*Passiflora edulis* Sims shell	[117]
2	Ruesgas-Ramón et al.	Identification and quantification of phytoprostanes and phytofurans of coffee and cocoa by- and co-products	Phytoprostanes (PhytoPs) and phytofurans (PhytoFs)	Biomarkers of oxidative stress in plants and humans. These compounds exhibit several interesting biological activities (e.g. neuroprotection and anti-inflammatory activities)	Liquid chromatography–tandem mass spectrometry	The contents of PhytoPs and PhytoFs in CP, CH, and CPH were, respectively, 654.6, 474.3 and 179.9, and 543.2, 278.0 and 393.8 ng per g dry weight (dw). The main PhytoP found in CP (171.37 ng per g dw) and CPH (37.12 ng per g dw) was 9-epi-9-F1t-PhytoP, while ent-9-L1t-PhytoP was the most abundant in CH (109.78 ng per g dw). The main PhytoF found in all sources was ent-16(RS)-13-epi-ST-Δ14-9-PhytoF, at 196.56, 126.22, and 207.57 ng per g dw in CP, CH, and CPH, respectively	Coffee pulp (CP), cocoa husk (CH) and cocoa pod husk (CPH)	[118]
3	Teixeira et al.	Early detection of lipid oxidation in infant milk formula by measuring free oxylipins—Comparison with hydroperoxide value and thiobarbituric acid reactive substance methods	Oxilipins	Oxylipins are effective in detecting early lipid oxidation and distinguishing between formulations containing different fatty acids.	UPLC-MS/MS	Several oxylipins increased in both formulas starting on day 7 (linoleic acid and alpha-linolenic acid-derived oxylipins in Formula 1 and DHA-derived oxylipins in Formula 2)	Infant milk formula	[119]
4	Samarra et al.	Analysis of oxylipins to differentiate between organic and conventional UHT milks	Oxylipins	These oxylipins could be promising as not only diet-dependent biomarkers for organic milk assessment	Chromatographic coupled to mass spectrometry	Several oxylipins (8-HEPE, 5-HEPE, 11-HEPE, 9-HEPE, 18-HEPE, 9-HOTrE, 13-HOTrE, 12,13-DiHODE and 15,16-DiHODE) could distinguish between organic and conventional milks	UHT milks	[120]
5	Dias et al.	Effects of industrial heat treatments on bovine milk oxylipins and conventional markers of lipid oxidation	Oxylipin	Assess the impact of milk processing	LC-MS	The results demonstrate that heat processing reduces milk oxylipin content and antioxidant capacity and that oxylipin and TAC measurements provide a new sensitive approach to assess the impact of milk processing on lipid oxidation	Raw bovine milk	[72]
6	Perez et al., 2020	Bioactive plant oxylipins-based lipidomics in eighty worldwide commercial dark chocolates: Effect of cocoa and fatty acid composition on their dietary burden	Phytoprostanes (PhytoPs) and phytofurans (PhytoFs)	As preventive agents against oxidative stress that could confer this dark chocolates an additional interest to consumers	UHPLC coupled to a triple quadrupole-MS/MS	The content of PhytoPs and PhytoFs in the array of chocolates analyzed oscillated from 2.45 to 1192.43 ng/g fw and from 144.57 to 840.19 ng/g fw, respectively.	Dark chocolates	[100]
7	Zhang et al.	Milk lipids characterization in relation to different heat treatments using lipidomics	Oxidized phosphatidylcholine, oxidized phosphatidylethanolamine, ether-linked phosphatidylethanolamine, diacylglycerol, triacylglycerol, and oxidized triacylglycerol	These biomarkers can potentially be used in the dairy industry to monitor the degree and method of heat treatment of milk	UPLC-Q-TOF-MS	Oxidized phosphatidylcholine, oxidized phosphatidylethanolamine, ether-linked phosphatidylethanolamine, diacylglycerol, triacylglycerol, and oxidized triacylglycerol can be used to differentiate raw, pasteurized, and ESL milk	Milk	[121]
8	Koch et al.	Comprehensive Analysis of Fatty Acid and Oxylipin Patterns in n3-PUFA Supplements	Oxylipins	Supplementing long-chain omega-3 polyunsaturated fatty acids (n3-PUFA) improves health	LC-MS/MS	Tthe oxylipin and fatty acid pattern allows gaining new insights into the origin and quality of n3-PUFA oils in supplements.	Food supplement	[122]
9	Teixeira et al.	Method optimization of oxylipin hydrolysis in nonprocessed bovine milk indicates that the majority of oxylipins are esterified	Oxylipins	In many biological matrices, the majority of oxylipins are bound (i.e. esterified), and a relatively small proportion (<10 %) exists in the free form.	Mass-spectrometry	The majority of bovine milk oxylipins are bound, and that linoleic-acid derived metabolites are the most abundant oxylipin species in free and bound lipid pools	Bovine milk	[123]

Lipidomics is an analytical method that uses the latest instruments including mass and tandem mass spectrometry, as well as liquid chromatography to perform comprehensive and efficient lipid analysis [50,51]. It integrates liquid chromatography and mass spectrometry methods to analyze lipids in biological samples and processed food products [52,53]. This method helps in simultaneously identifying and quantifying hundreds to thousands of lipid species in a given sample [54]. In addition, it facilitates comprehension of lipid metabolism and the implications.

Several oxylipins categories, such as prostaglandins, leukotrienes, and epoxy-fatty acids, may be accurately determined and measured in biological samples and dietary matrices using lipidomics method [55,56]. The characterization of oxylipins profile in various circumstances provides valuable insights into the functions in relation to health and disease, specifically in identifying potential biomarkers or therapeutic targets. Moreover, lipidomics contributes to the development of healthier and more stable food options by delineating lipid composition changes that occur during food processing [56]. The integration with other omics disciplines offers new dimensions of lipid metabolism and the impact on health and nutrition, driving advancements in both fundamental science and practical applications.

### 2.3 Significance of lipidomics in advancing studies on processed food products

Lipidomics offers a full understanding of lipid composition and alterations occurring during food processing [[57], [58], [59], [60]]. According to Wang et al. [4], lipids have significant implications in the determination of sensory characteristics, nutritional value, and longevity of processed food products. It is important to comprehend lipid composition of products to enhance the formulation, guarantee food safety, and fulfill consumer expectations for healthier and environmentally friendly food choices.

Generally, lipidomics presents numerous fundamental advantages making it a highly desirable instrument in the investigation of processed food. The extensive analysis and quantification of various lipid species in complex food matrices allows for a thorough examination of lipidome in processed foods. Understanding this information is crucial for evaluating the nutritional composition and possible health consequences of various lipid constituents. Additionally, lipidomics assumes a crucial function in the surveillance of lipid oxidation due to the influence on sensory attributes, shelf life, and nutritional value [61]. The examination of the production and durability of oxidation byproducts, such as oxylipins provides an approach to alleviate lipid oxidation and prolong the longevity of processed food items [62,63]. Lipidomics also plays a crucial role in facilitating the advancement of functional foods by the identification and quantification of bioactive lipids possessing distinct health-promoting properties. This empowers the food sector to produce goods that are fortified with advantageous lipid constituents.

## 3 The formation of oxylipins in processed foods

### 3.1 Mechanisms of lipid oxidation and the generation of oxylipins

The presence of oxylipins in processed meals is a multifaceted phenomenon that originates from the oxidative breakdown of PUFAs including LA, AA, and DHA, frequently found in a variety of food sources, such as vegetable oils, nuts, and fish [64,65]. During food production and storage, PUFAs are exposed to various conditions, potentially resulting in lipid oxidation [66,67] and the synthesis of oxylipins [68].

**Fig. 2:**
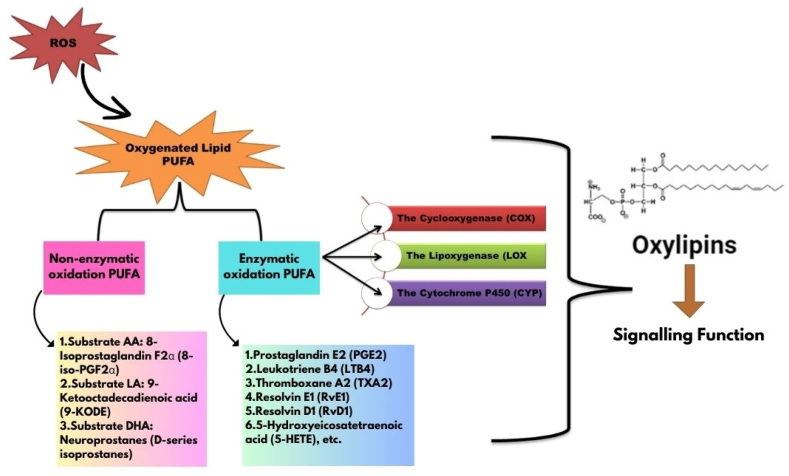
The general Process of oxylipins formation in food products. Ros: Reactive oxygen species; PUFA: Polyunsaturated fatty Acids; AA: Arachidonic Acid; LA: Linoleic acid; DHA: Docosahexanoic acid.

The primary mechanisms of oxylipins production in processed food products are enzymatic and non-enzymatic oxidation reactions (Fig. 2). The catalytic activity of enzymes, such as COX, LOX, and cytochrome P450 (CYP), is referred to as enzymatic oxidation. According to Barbosa et al. [69], these enzymes aid in the process of oxygenating certain carbon atoms in PUFAs backbone, forming oxylipins with distinct chemical structures and biological characteristics. Various physiological processes in food processing are significantly regulated by enzyme pathways.

Non-enzymatic oxidations are defined by reactions mediated through free radicals, which can be triggered by heat, light, or metal ions [70]. The oxidative mechanisms result in the peroxidation of PUFAs, leading to the formation of several reactive lipid compounds such as hydroperoxides, epoxy-fatty acids, and isoprostanes. The reactive intermediates have the potential to experience subsequent rearrangements and fragmentation processes, playing a role in the generation of a wide spectrum of oxylipins commonly observed in processed food products [71].

The presence of oxylipins in processed foods has important consequences for the overall quality of products, due to the potential to introduce undesirable odors, promote rancidity, and diminish the nutritious content [45,72,73]. Moreover, the existence of prostaglandins and leukotrienes may have potential inflammatory consequences or associations with human well-being [15,32].

### 3.2 Factors influencing lipid oxidation in processed food products

Lipid oxidation in processed food products is a multifaceted phenomenon impacted by various significant elements. The degree of lipid susceptibility to oxidation is influenced by the presence of PUFAs, which have heightened vulnerability to oxidation due to the presence of many double bonds [74]. Moreover, many conditions, including temperature, exposure to oxygen, and light, have the potential to expedite the process of lipid oxidation [75,76]. For example, temperature can cause damage to the structure of lipids, leading to oxidation of the compound. All processing steps, including raw product selection, harvesting, storage, refining, manufacturing, and distribution, have a significant impact on the quality of lipids in the finished product. This acceleration triggers a cascade of events that ultimately culminate in the generation of lipid radicals [77]. Pro-oxidants, including transition metal ions and pro-oxidant enzymes, can function as catalysts, facilitating the process of lipid oxidation [76,78]. Furthermore, the management of lipid oxidation is influenced by several factors, including water activity, antioxidants, and packaging choices [[79], [80], [81]].

## 4 Analytical methods in studying oxylipins in processed foods

**Fig. 3:**
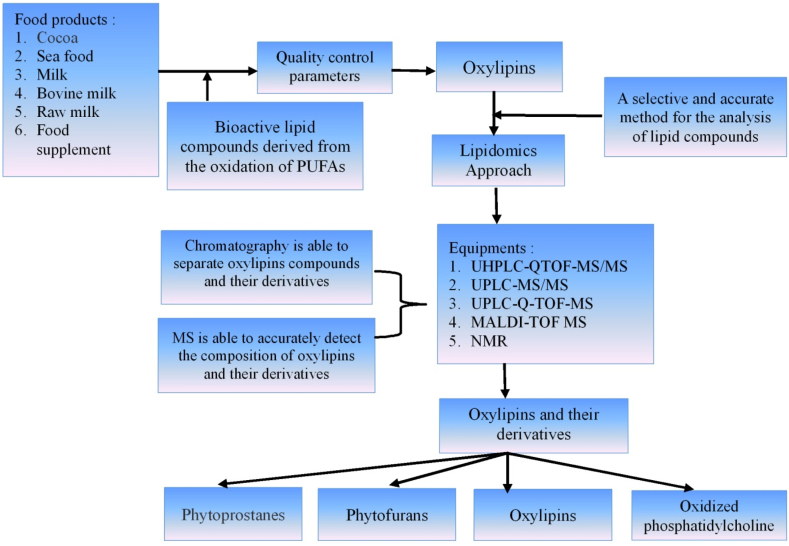
Oxylipins and the derivatives analysis in food quality control. PUFAs: Polyunsaturated fatty acids; UHPLC-QTOF-MS/MS: Ultra-high performance liquid chromatography-Quadrupole time-of-Flight tandem mass spectrometry; UPLC-MS/MS: Ultra-performance liquid chromatography-tandem mass spectrometry; UPLC-Q-TOF-MS: Ultra-high performance liquid chromatography with quadrupole time-of-Flight mass spectrometry; MALDI-TOF MS: Matrix-Assisted Laser Desorption Ionization–time of Flight mass spectrometry; NMR: Nuclear magnetic resonance.

As shown in Fig. 3, analytical methods are crucial for examining oxylipins in processed food products due to the ability to easily identify, measure, and characterize biologically active lipid molecules. Considering oxylipins are rare and have variable structures, analysis requires sensitive and specific methods. Using mass spectrometry (MS), oxylipins can be identified and quantified by examining the molecular mass and fragmentation patterns. Furthermore, gas chromatography-mass spectrometry (GC-MS) and liquid chromatography-mass spectrometry (LC-MS) are often used as reported by Koelmel et al. [82] and Zhang et al. [44]. Liquid chromatography (LC) and MS are frequently used to identify and separate oxylipins present in complex food matrices. In this context, ultra-high-performance liquid chromatography (UHPLC) and high-performance liquid chromatography (HPLC) methods are commonly used to provide increased resolution and sensitivity. Furthermore, nuclear magnetic resonance (NMR) spectroscopy is used to determine the structure and amount of oxylipins. This method has the advantages of good sensitivity and selectivity while the disadvantages include complex procedures and high cost [83]. The enzyme-linked immunosorbent test (ELISA) allows for feasible and accurate quantification of specific subclasses or biomarkers [12]. Moreover, the Stable Isotope Dilution Assay (SIDA) is a quantitative method with great precision and accuracy in quantifying oxylipins by using isotopically labeled standards. It is characterized by improved consistency and reliability in the collection of quantitative data using isotopically labeled standards that show a high degree of resemblance to the target [84].

Advanced analytical methods including high-resolution mass spectrometry (HRMS) and high-resolution liquid chromatography (HRLC) facilitate the study of complex oxylipins combinations [85,86]. The combined application of HRMS with HPLC or UHPLC has led to improved detection through higher separation capabilities and decreased interference from co-eluting chemicals [87,88]. The integration of sophisticated methods facilitates an exhaustive and dependable characterization of oxylipins in a variety of biological and nutritional contexts.

## 5 Challenges and Limitations in analyzing oxylipins in complex food matrices

Several difficulties and restrictions are associated with the characterization of oxylipins in complex food matrices due to the diverse spectrum of lipids and chemicals found in the samples. In this context, a significant obstacle is the low concentration of oxylipins in processed foods, making the detection and quantification difficult. According to Chen et al. [89], precise identification requires the use of highly sensitive analytical methods due to the frequent presence in trace levels. The structural variation among oxylipins is also a challenge, necessitating the use of unique analytical methods for recognition and measurement [90,91].

Interferences during the analysis process may occur due to the intricate structure of food matrices [92]. Food samples include a wide variety of lipid classes, proteins, carbohydrates, and other components that make the detection of oxylipins difficult, particularly affecting the precision and selectivity of the results. The process of preparing food samples for analysis underscores the implementation of extraction and purification techniques. However, these steps may lead to the loss or modification of oxylipins, potentially compromising the accuracy and dependability of the analytical results [93].

The absence of standardized techniques for the analysis of oxylipins in food matrices adds to the complexity of comparing and interpreting results across various studies. Diverse analytical platforms, sample preparation processes, and calibration standards used by various laboratories can introduce discrepancies in results, impeding the attainment of uniform and replicable data [93].

An additional constraint is the possible alteration of oxylipins during the processes of food manipulation and preservation [67,80,94]. Oxylipins are prone to oxidation and breakdown in diverse circumstances, leading to modifications in quantities and composition within processed food items. The interpretation of results and comprehension of oxylipins profile in processed meals underscores the meticulous examination of this dynamic activity.

## 6 Impact of oxylipins on processed food quality

Oxylipins have a substantial and multifaceted impact on the quality of processed foods, influencing the flavor, aroma, nutritional content, and shelf life [77,95]. Certain oxylipins, such as volatile aldehydes and ketones, have been connected to unpleasant food product odors and flavors [96,97]. Unfavorable sensory attributes may lead to consumer dissatisfaction and a reduction in the acceptance of the product [98]. Moreover, specific oxylipins generated in lipid oxidation procedures, namely hydroperoxides and secondary oxidation products, might be detrimental to the nutritional value of processed foods [95] due to the degradation of vital nutrients.

## 7 Biological activities of oxylipins and potential health effects

Oxylipins play crucial roles in lipid metabolism and energy regulation, with specific types such as endocannabinoid derivatives, participating in appetite control, energy balance, and glucose metabolism [99], while dysregulation may contribute to obesity and metabolic disorders. Furthermore, oxylipins are implicated in oxidative stress [100,101] with isoprostanes, serving as markers of lipid peroxidation and oxidative damage [38].

The diverse biological activities of oxylipins have significant health effects on the human body. Some lipid oxidation products not only impact the quality of food but also have implications for various diseases and human health. Oxidative stress in the body refers to an imbalance between oxidants and antioxidants, where oxidants are favored in biological systems. Although the body possesses defensive enzymes and antioxidant compounds, numerous sources of oxidants or free radicals can disrupt the equilibrium. The role of oxylipins in inflammatory processes is considered very crucial. For example, prostaglandins and leukotrienes play a crucial role in inflammation by serving as mediators that regulate immune responses and facilitate the regeneration of tissues [31,102]. Macrophages have a significant role in the defense against pathogens, facilitation of tissue repair, and initiation of immunological responses in the context of injuries or infections.

Although oxylipins have both beneficial and detrimental health effects, the overall impact depends on the type, concentration, and context in the body. Maintaining a balance of pro-inflammatory and anti-inflammatory oxylipins is crucial for proper immune function and overall health. Moreover, understanding the biological functions can yield significant knowledge on prospective utility as therapeutic targets [20] in the treatment of inflammation-related disorders and the production of functional foods containing beneficial oxylipins. Further investigation is required to gain a comprehensive understanding of the intricate interplay and physiological impacts of these bioactive lipid mediators.

The presence of oxylipins, particularly those with pro-inflammatory properties, may have implications for food safety and consumer health. Chronic inflammation from the consumption of processed foods containing pro-inflammatory oxylipins may contribute to the development of various chronic diseases, including cardiovascular disease and certain types of cancers [103].

Some oxylipins, such as certain epoxy-fatty and hydroxy-fatty acids, have potential health benefits, showcasing anti-inflammatory and cardioprotective effects [55]. In this context, the presence of specific beneficial oxylipins in processed foods could contribute to the development of functional food products with health-promoting properties.

## 8 Association between oxylipins in processed foods and chronic diseases

Processed foods containing PUFAs are susceptible to lipid oxidation when exposed to heat. This oxidation process leads to the production of harmful oxylipins with pro-inflammatory properties. For example, certain prostaglandins and leukotrienes can promote chronic inflammation when consumed in excess [104], while some epoxy-fatty acids have vasodilatory effects, contributing to the regulation of blood pressure and vascular function [105]. Epoxy-fatty acids may also impact platelet aggregation and thrombosis, potentially influencing cardiovascular disease risk.

The presence of harmful oxylipins in processed foods can lead to oxidative stress, a condition characterized by an imbalance between reactive oxygen species and antioxidant defense systems in the body. Oxidative stress is associated with cellular damage and the development of various chronic diseases, including neurodegenerative disorders and cardiovascular diseases [94,106].

## 9 Mitigating oxylipins formation in processed foods

### 9.1 Antioxidants and roles in preventing lipid oxidation and oxylipins generation

To preserve product quality, safety, and consumer health, it is important to mitigate oxylipins formation in processed foods. Several strategies can be used to minimize the generation of detrimental oxylipins during food processing and storage. A primary method entails the use of antioxidants. As stated by Budilarto and Kamal-Eldin [107] and Domínguez et al. [25], antioxidants efficiently scavenge and neutralize lipid radicals, disrupting the cascade of lipid oxidation and impeding the production of detrimental oxylipins. Therefore, antioxidants can effectively reduce the production of harmful oxylipins, inhibiting lipid peroxidation of PUFAs, in processed meals [108].

The use of natural antioxidants, such as vitamin E, vitamin C, and polyphenols derived from fruits, vegetables, and specific spices, is a prevalent practice in the incorporation of processed food formulations to augment oxidative stability [109,110]. The antioxidants function as sacrificial agents that effectively counteract the harmful effects of free radicals, thereby inhibiting the onset and progression of lipid oxidation. According to Konfo et al. [111], the addition of natural antioxidants in processed foods plays a crucial role in preserving the nutritional quality and sensory characteristics of PUFAs.

Antioxidants, including both naturally occurring such as vitamin E and manufactured ones, play a crucial role in the process of neutralizing lipid radicals and decelerating oxidation events. The implementation of appropriate packaging and storage conditions is crucial to minimize lipid oxidation and maintain the quality, nutritional composition, as well as longevity of processed foods [81]. This entails using packaging materials that possess effective oxygen and light barrier properties, as well as storing the foods in cool and dry environments. The comprehension and regulation of these variables are crucial in guaranteeing consumer contentment, security, and the manufacturing of superior processed food products.

In conjunction with naturally occurring antioxidants, the food industry frequently uses synthetic antioxidants, namely butylated hydroxyanisole (BHA) and butylated hydroxytoluene (BHT), to prolong the shelf-life of processed food items through the inhibition of lipid oxidation [112]. Although there have been concerns regarding the use, synthetic antioxidants continue to be authorized for particular applications, contingent upon adherence to regulatory requirements and safety evaluations [113].

To preserve product quality and safety, it is essential to control the formation of detrimental oxylipins in processed foods. In this context, applicable strategies include the addition of antioxidants [45], the optimization of processing conditions, and the use of appropriate packaging materials.

### 9.2 Strategies for minimizing oxylipins content in processed food products

The reduction of oxylipins production in processed meals requires the use of many measures aimed at minimizing the presence of these biologically active lipid molecules. In this context, a critical method entails the identification of suitable lipid sources. The use of oils and fats containing elevated levels of saturated fatty acids or MUFAs, which possess greater oxidation resistance [12,114], has the potential to decrease the vulnerability to oxylipins production. Additionally, implementing certain strategies, such as the use of antioxidants [25] or optimizing storage conditions, has demonstrated the potential to reduce the possibility of lipid oxidation risks. These measures, in general, might help decrease the synthesis of oxylipins.

### 9.3 Importance of optimizing processing conditions to reduce oxylipins formation

The optimization of processing conditions is crucial to reduce the production of oxylipins in processed foods. During food processing, various parameters including temperature, duration, and exposure to light and oxygen play a crucial role in determining the degree of lipid oxidation and subsequent production of oxylipins. As stated by Del Caño-Ochoa et al. [115], the presence of high temperatures and prolonged processing times hasten the oxidation of lipids, leading to the production of oxylipins. Therefore, it is possible to effectively reduce the risk of lipid oxidation and the production of oxylipins by implementing lower processing temperatures and shorter processing times [82].

According to Lobo et al. [108], exposure to light and ambient oxygen produces free radicals, which are the starting point of oxidative processes. Processed foods may be protected against the effects of air and light by using packaging materials that provide an adequate barrier against these environmental factors. This preventive strategy lowers the amount of oxylipins while preserving the stability and quality of lipids. Furthermore, using inert environments during the processing phase can successfully prevent lipid oxidation and oxylipins. Lipid oxidation is significantly reduced when an oxygen-depleted environment is used, as evidenced by the results of Lee et al. [116]. This safeguarding measure effectively preserves lipid composition and the general quality of food products.

## 10 Conclusion and future perspectives

In conclusion, prospectively studying oxylipins and processed foods including a thorough examination of many categories and functions in various food matrices, offers numerous opportunities to shape the future of the industry. In particular, comprehensively assessing the influence on flavor, scent, and nutritional composition holds significant potential for further product development. Evaluating the effects of processing techniques and storage circumstances can provide valuable insights into approaches aimed at reducing detrimental oxylipins and improving the shelf life as well as safety of food products.

The incorporation of lipidomics into the domain of food quality control and safety measures marks a significant progression, as it facilitates the contemporaneous assessment of oxylipins levels and lipid oxidation in processed food products. Implementing advanced analytical methods, such as mass spectrometry and nuclear magnetic resonance in routine food quality assessments, can enhance product traceability and ensure compliance with safety standards. These measures safeguard consumers from potential health risks associated with detrimental oxylipins in processed foods.

Oxylipins studies present exciting potential applications in the industry, offering opportunities for the development of functional foods with targeted health benefits. By understanding the roles of specific oxylipins in inflammation modulation and lipid metabolism, food manufacturers can design products that promote wellness and reduce the risk of chronic diseases. Additionally, the incorporation of natural antioxidants into processed foods may be a novel approach to enhance nutritional value and appeal to health-conscious consumers.

Future perspectives in oxylipins studies as well as processed foods are promising. Continued collaboration between food scientists, lipidomics studies, and nutritionists will drive innovation and deepen the current understanding of the complex interactions between oxylipins and food components. Further studies exploring the effects of processing methods, novel ingredients, and innovative packaging techniques on oxylipins formation will facilitate the development of healthier and more stable processed food products. As technology and analytical tools advance, the integration of lipidomics into food industry practices is expected to become increasingly feasible, allowing for more precise monitoring and optimization of processed food quality. Furthermore, ongoing investigations into processed foods foster the development of innovative, nutritious, and safer food choices, catering to the evolving demands of consumers related to healthier and sustainable dietary options.

## Fundings

The work was funded by Grant of Ministry of Education, Culture, Research and Technology (MoECRT), Republic of Indonesia [grant number: 3969/UN6.3.1/PT.00/2024]. And the APC was supported by 10.13039/501100015690Universitas Padjadjaran, Indonesia.

## Institutional review board statement

Not applicable.

## Informed consent statement

Not applicable.

## Data availability statement

Data will be made available on reasonable request.

## CRediT authorship contribution statement

**Putri Widyanti Harlina:** Writing – review & editing, Writing – original draft, Visualization, Validation, Supervision, Resources, Project administration, Funding acquisition, Formal analysis, Data curation, Conceptualization. **Vevi Maritha:** Writing – original draft, Methodology, Investigation, Formal analysis, Data curation. **Xiang Yang:** Writing – review & editing, Validation, Supervision. **Roy Dixon:** Writing – review & editing, Validation, Supervision. **Muchtaridi Muchtaridi:** Writing – review & editing, Validation, Supervision. **Raheel Shahzad:** Methodology, Investigation, Writing – review & editing. **Ernisa Adha Nur’Isma:** Methodology, Investigation.

## Declaration of competing interest

The authors declare that they have no known competing financial interests or personal relationships that could have appeared to influence the work reported in this paper.
